# Social rejection sensitivity and its role in anorexia nervosa: a systematic review of experimental literature

**DOI:** 10.1186/s40337-025-01261-7

**Published:** 2025-07-10

**Authors:** Senan Coughlan-Hopkins, Cristina Martinelli

**Affiliations:** 1 Department of Psychology, City St George’s, University of London, London, UK; 2 Department of Psychology, Kingston University, London, UK

**Keywords:** Anorexia nervosa, Eating disorders, Social rejection, Social exclusion, Attentional bias, Need to belong, Interpersonal functioning

## Abstract

**Objective:**

Social rejection sensitivity (SRS) is characterised by anxious expectations of rejection, and the increased tendency to readily perceive and react intensely to rejection-based cues. It has been suggested SRS may play a role in anorexia nervosa (AN). Our review investigates whether SRS is exhibited in AN, and the cognitive mechanisms that underly this disposition.

**Method:**

We included experimental studies if they used social threat or rejection-based stimuli, reported on measures related to either cognitive, emotional, and/or behavioural responses, and compared patients with a diagnosis of AN and/or those who have recovered from the illness with healthy controls.

**Results:**

This article identified 47 eligible studies, with risk of bias assessment indicating the research was of good quality. Main findings showed patients with AN exhibit attentional bias towards social rejection cues, negative interpretation bias during ambiguous social scenarios, and heightened negative affect during and following rejection-based experiences. Physiological blunting during and following rejection-based experiences was observed in AN with some evidence to suggest this remediates during the process of weight-restoration. demonstrating an incongruence between affective and somatic experience in active illness.

**Discussion:**

Our results suggest females with AN display a cognitive profile that could lead to a tendency to expect rejection, readily perceive rejection and react with more intense negative affect to rejection-based cues, with limited evidence to suggest this cognitive profile persists in recovery. Our results can be interpreted through theoretical models that postulate drive for thinness may partially function to cope with anticipated or experienced rejection.

**Supplementary Information:**

The online version contains supplementary material available at 10.1186/s40337-025-01261-7.

## Plain English Summary

Social rejection is an unpleasant experience for most; however, some people may be more sensitive to rejection than others. This trait defined as social rejection sensitivity has been suggested to be an important feature of AN. However, the ways in which social rejection sensitivity may contribute to AN is incompletely understood. We found that individuals with AN were more likely to attend to social cues that signal rejection and were more likely to interpret ambiguous social scenarios in a negative manner. AN patients experienced heightened negative emotions and reduced physiological responses during stressful social situations.

## Introduction

Anorexia Nervosa (AN) is an eating disorder (ED) characterised by severe calorie restriction, an intense fear of gaining weight, and distortions of body image [1]. Recent literature points towards dysfunctions of social cognition bearing important clinical implications [2–4].

A growing literature has explored social rejection sensitivity (SRS), namely the tendency to anxiously expect, readily perceive, and react intensely to cues of social rejection [5]. Theoretical models have proposed SRS develops because of a combination of biological-based factors that amplify negative affect [6], and early experiences of rejection, such as those characterising insecure attachment, bullying, or trauma [6]. This is assumed to contribute to a pathway resulting in cognitive biases [7], often driven by attentional [8], and inferential processing [9], that can lead to individuals readily perceiving benign or neutral cues as evidence of rejectionn [7]. With repeated experiences of perceived or actual rejection, SRS can increase over time, resulting in reduced capacity for emotional regulation and further hypersensitivity to cues of rejection [6].

Within this context, the pursuit of thinness may function to pre-emptively cope with the fear of rejection by maintaining a sense of control over one’s body and appearance [10]. Similarly, experiencing rejection may trigger restrictive eating, to numb or reduce the intensity of emotional distress elicited [11]. This aligns with the ‘cognitive-interpersonal’ model [12] which postulates chronic starvation may inhibit affective experience, whilst concomitantly remedying social rejection by fostering a sense of belonging, because of others’ reactions of interest, sympathy and care to one’s emaciation and unhealthy appearance. This may further explain why patients value aspects of their illness [13] which often presents as a barrier to recovery [13].

In support of the above, AN patients experience significant socio-emotional and interpersonal difficulties [12, 14], with social stressors often precipitating AN onset [10, 15]. Early experiences that shape SRS such as bullying, weight and shape teasing have also been associated with ED psychopathology [16, 17] and a recent meta-analysis [18] has shown higher rates of attachment insecurity in AN, including fear of abandonment, which can be considered a manifestation of a rejection-based experience. Together, this points to a possible link between early adversity, SRS and ED psychopathology in AN. With this proposition strengthened by a small number of cross-sectional findings that have consistently shown adolescent [19, 20] and adult AN patients [21, 22] score high on self-reported measures of SRS [23–25].

Experimental paradigms used to study SRS investigate several domains of cognitive processing. This includes the examination of attentional processing towards social threat information, typically words or pictures of faces that can signal anticipated rejection. Other studies focus on social interpretation, relying on socially ambiguous information to capture rejection-based inferences in social contexts. Memory tasks have been used to investigate recollection biases linked to social and non-social stimuli, while emotional recognition tasks are used to measure the accurate identification of emotions that carry actual or potential social threat. Further studies induce social stress, exclusion or rejection to investigate emotional, behavioural and physiological responses. For conceptual precision, we distinguish social threat processing which refers to the anticipation of social danger or harm, from social rejection; the subjective experience that follows, regardless of whether social rejection is real or imagined. This terminology framework reflects our conceptualisation of SRS as encompassing both anticipatory and post-exposure reactivity mechanisms.

To date, only one paper [26] has systematically investigated features of social cognitive processing in relation to interpersonal stress in EDs. Through a series of meta-analyses, authors found heightened attentional bias (AB) towards social threat cues, negative interpretations of social scenarios, lower heart rate after exposure to interpersonal stress, and greater negative affect before and after interpersonal stress. However, this systematic review combined samples from different diagnostic ED groups, which may have biased findings, and leaves us wondering the extent to which SRS is systematically observed in AN. Lastly, the study only included papers up to April 2017. Our review aims to fill these gaps by focusing exclusively on AN and the unique clinical characteristics that contribute to SRS in this illness. From synthesising the evidence base of experimental research in this area we aim to answer the following questions: 1) Are people with AN more sensitive to experiences of social rejection or social threat compared with healthy controls? 2) What are the underlying cognitive systems that propel SRS in AN? 3) Is SRS a state (i.e., only occurring in the acute phase of the illness) or trait characteristic of AN?

## Methods

This review was conducted in accordance with the Preferred Reporting Items for Systematic Reviews and Meta-Analyses (PRISMA) guidelines [27]. The study is registered with PROSPERO, CRD42023382697. Note that our pre-registration included a fourth research question aimed at investigating whether SRS is linked to specific symptoms or clinical features of AN. However, this component has been excluded from the manuscript due to the heterogeneity of the associations observed in the literature, which precluded meaningful synthesis and interpretation of findings.

### Literature search

The electronic databases OVID; PsychINFO, MEDLINE, and Embase were searched using the following search string:"social*"OR social exclusion*"OR"ostracis*"or"ostracize*"OR"social punish*"OR"social harm*"OR"social sensitiv*"OR"social reject*"or"reject OR"cyberball task"OR"critical feedback") AND (diet* restrain*"OR"diet* restrict*"OR"anorexi*"). All stated databases were searched from database inception to July 2024, limiting search to the English language and studies human subjects. Bibliographies of key articles were also inspected.

### Eligibility criteria

Experimental studies were included if they: i) utilised social threat or rejection stimuli; ii) reported on measures related to either cognitive, emotional, and/or behavioural response; iii) compared samples of patients with a diagnosis of AN and/or those who have recovered from AN (RecAN), with a sample of healthy controls (HCs).

### Study selection and data collection

Titles and abstracts were first reviewed against eligibility criteria, followed by full text-articles. Screening was independently performed by two researchers, where there were disagreements on eligibility, studies were re-evaluated by a third researcher and a discussion took place.

## Search selection

### Quality assessment of included studies

Risk of bias was assessed with an adapted version of the Newcastle–Ottawa Scale for case control studies [28] (NOS; Supplementary materials 1). This questionnaire focuses on three dimensions, i) selection, which assesses whether the definition of clinical cases was representative of AN and RecAN, and whether HCs were recruited without a current psychiatric diagnosis or impairment that would compromise the integrity of findings, ii) comparability, assessing whether the study accounted for important factors, such as controlling for age, BMI, IQ, depression, and anxiety, iii) exposure, which assesses the standardisation of experimental exposure. The NOS is based on a scoring system indicating low (0–3), moderate (4–5), good (6–7) or excellent (8–9) quality [28]. Two researchers independently rated all the studies. Where there was a discrepancy in scoring, a third reviewer mediated the final decision through a discussion.

### Data extraction and synthesis

The following data from eligible studies was extracted where reported: 1) publication details including author(s), publication date, and country; 2) study information: study setting, and design, the experimental task used, and other study measures of importance (e.g., anxiety and depression); participant characteristics: sample size, sex, age, key demographics, illness duration, BMI, average number of hospitalisations, average length of illness, psychiatric co-morbidities, and the use of psychiatric medication; 4) study results: the findings in relation to the primary outcomes. Data synthesis followed the Synthesis Without Meta-Analysis reporting guidelines [29] (SWiM), with our results primarily structured according to the cognitive systems investigated in relation to SRS in AN. As we found methodological diversity, we further organised the results by experimental paradigms to enable comparisons between tasks used.

## Results

**Fig. 1:**
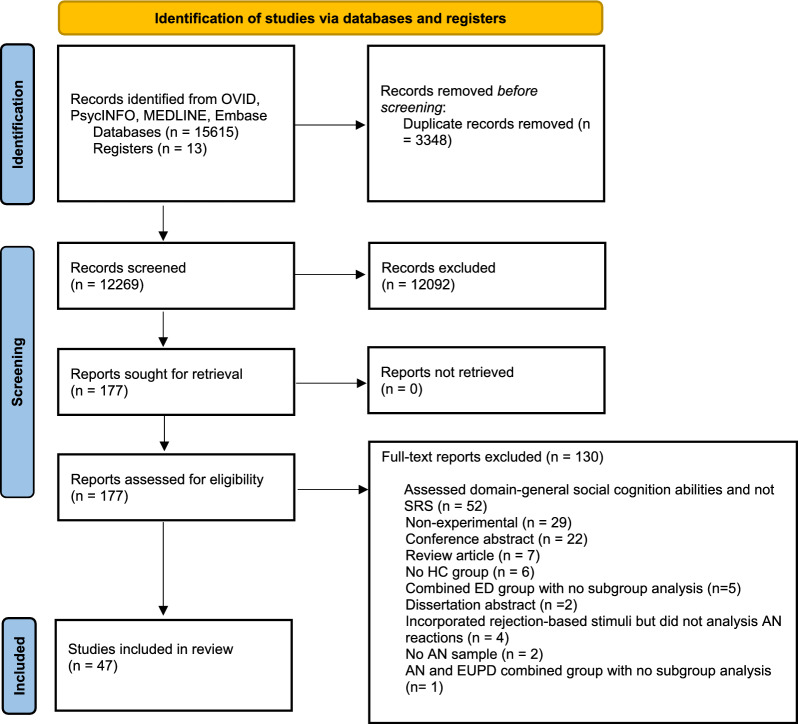
PRISMA flow diagram of the screening process

The initial search generated 15,615 studies, with an additional 13 studies identified from the bibliographies of key papers and review articles. We removed 3348 duplicates, leaving 12,269 studies screened for eligibility, of which 12,092 were excluded based on title or abstract inspection. This left 177 studies with full texts assessed for eligibility, of which 130 studies were excluded, resulting in 47 papers being included. The PRISMA flowchart (Fig. 1) shows the inclusion process and details the reasons for exclusion.

### Countries of research

The research was conducted across 14 countries, with the majority of studies (85%) taking place in Europe; Germany (12), United Kingdom (12), Italy (7), Belgium (2), Spain (2), France (1), Netherlands (1), Norway (1), Poland (1), Germany and Switzerland multicentre (1). The remaining studies were conducted in United States of America (3), South Korea (2), Australia (1) and Israel (1).

### Study characteristics

A total of 47 studies were included in the current systematic review categorised into five cognitive systems. These were social attention (14/47 papers), social inference (2/47 papers), social memory (3/47 papers) emotional recognition and regulation (13/47 papers), and behavioural, affective, and physiological responses (15/47 papers). Two studies incorporated experimental paradigms across two of the aforementioned areas.

### Recruitment and sample characteristics

A total of 3375 participants were recruited across studies, of which 1458 (43.2%) were currently ill with AN, 87 (2.57%) were RecAN, and 1847 (54.7%) were HCs. One study based the AN diagnosis of ICD-10 criteria (1/47), while the rest used DSM-III (3/47) DSM-III-R (1/47), DSM-IV (22/47), DSM-IV-R (4/47), DSM-V (16/47). The Structured Clinical Interview for DSM (SCID; [30] was the most frequently used tool to assessment clinical diagnosis. Eleven studies did not disclose the assessment tool to obtain diagnosis. The most common exclusion criteria for HCs were self-reported current or historical diagnosis of Axis 1 psychiatric disorder.

In the majority of studies RecAN [31–33] had a lifetime diagnosis of AN, BMI above 18.5 for at least a year, and scored below clinical threshold scores on the Eating Disorder Examination Questionnaire (EDE-Q) [34], one study defined recovery as medically stable and partially or fully weight-restored [35]. Recruitment occurred in hospital inpatient services (12/45) [36–47], community settings (11/45) [31, 32, 48–56], and a mixture of the two (6/45) [33, 57–61], with 18 studies [35, 62–78] providing no information regarding setting of recruitment. HC status was formally assessed via clinical screening interviews in 20 studies only. HCs were more commonly excluded if reporting a psychiatric illness and body weight below healthy standards. Only 9 studies reported on co-morbidities of the AN sample, which included a subset of participants with depression or anxiety (8/9) [39, 55, 63, 64, 68, 70, 71, 77] obsessive–compulsive disorder and social anxiety disorder (SAD; 1/9) [38] or SAD only (2/9) [55, 77].AN participants consistently scored higher than HCs on self-report of ED psychopathology, depression and anxiety. Thirty studies recruited adult participants, 8 [35, 44, 64, 67, 68, 70, 74, 77] recruited children and adolescents, and 9 [32, 33, 37, 48, 52, 56, 63, 66, 75] recruited children, adolescents and adults. Only 7/47 studies [38, 45–47, 50, 61, 62] recruited male participants, most of which had a female majority sample, with only one study [62] recruiting all males. Only 8 studies [33, 35, 38, 40, 48, 57, 62, 72, 79] reported the ethnicity of AN patients, with all these studies recruiting ≥ 87% white samples.

### Risk of bias

The mean NOS assessment for all studies was 6.47, indicating good quality publications (see outcome tables 1. for a breakdown of NOS scores). Risk of bias assessment was further calculated for each subsection, revealing good quality for social attention (mean = 6.8), social inference (mean = 6), social memory (mean = 6.33), emotional recognition and regulation (mean = 6.85), and moderate quality for affective, behavioural and physiological responses (mean = 5.73).

**Table 1:** Description of social attention experimental paradigms, study outcomes and risk of bias

Author and Date	Country	Participants	Age	BMI	Experimental paradigm	Outcome measure	Main findings	Risk of Bias
			M SD	M SD				
Bang et al., [31]	Norway	RecAN = 22 HCs = 21	27.3 $\pm$ 5.14 26.0 $\pm$ 4.71	20.39 $\pm$ 1.66 21.85 $\pm$ 1.76	Dot-probe Task	Attentional bias towards angry faces vs. neutral faces	No differences between groups	7
Cardi et al., [32]	United Kingdom	AN = 29 RecAN = 13 HCs = 50	*n/s*	*n/s*	Dot-probe Task	Attentional bias towards neutral and rejecting poses, and neutral and accepting poses	AN and RecAN participants displayed a significant AB towards rejection poses, and delayed disengagement from rejection poses. Self-reported early experiences of adversity predicted heightened vigilance to rejection poses	5
Cserjési et al., [36]	Belgium	AN-R = 33 HCs = 63	21.8 $\pm$ 3.4 22.1 $\pm$ 3.9	14.8 $\pm$ 1.3 20.4 $\pm$ 1.4	Affective Priming Task	Schematic faces depicting neutral, positive (happiness), and negative (anger, sadness) primes before being replaced by positively and negatively valanced adjectives (e.g., hostile). Prime facilitation effects were observed between congruent (e.g., negative prime – negative target) vs. incongruent trials (e.g., negative prime – positive target)	AN participants recorded faster RTs for negative congruent trials vs HCs, and slower RTs for incongruent (negative prime – positive target word) trials vs. HCs	8
Gilon Mann et al., [37]	Israel	AN = 55 HCs = 19	$18. 43\pm$ 3.37 18.48 $\pm$ 3.5	16.09 ± 2.35 19.9 ± 1.73	Dot-probe Task	Attentional bias towards eating disorder threat words (e.g., “FAT”) vs. general and social threat combined (e.g., “GUILT”) vs. neutral words	Within (AN-R vs. AN-BP) group differences observed. AN-R displayed an attentional bias towards all stimulus types. AN-BP showed an attentional avoidance towards all stimuli type. Between-group differences showed no attentional avoidance or bias towards all stimulus types in HCs	7
Goddard et al., [62]	United Kingdom	AN = 14 HCs = 42	*n/s*	*n/s*	Emotional Stroop	Attentional bias towards angry vs. neutral faces	No AB between male AN patients and male HCs was observed	7
Harrison et al., [33]	United Kingdom	AN = 50 HCs = 90 (AN-R = 35 AN-BP = 15)	26.7 $\pm$ 9.82 28.50 $\pm$ 9.93	15.38 $\pm$ 1.83 21.61 $\pm$ 1.89	Emotional Stroop	Attentional bias towards angry vs. neutral faces	Greater attentional bias for angry faces vs. neutral faces in AN compared to HCs. No within (AN subtype) group differences observed	7
Harrison et al., [57]	United Kingdom	AN = 50 RecAN = 35 HCs = 90	26.7 $\pm$ 9.82 29.00 $\pm$ 10.62	15.38 ± 1.83 21.15 $\pm$ 21.61	Emotional Stroop	Attentional bias towards angry vs. neutral faces	Greater attentional bias for angry faces in AN and RecAN compared to HCs. No between (AN vs. RecAN) group differences observed	8
Kanakam et al., [48]	United Kingdom	*n/s*	*n/s*	*n/s*	Emotional Stroop	Attentional bias towards angry vs. neutral faces	Twins with AN had a significantly greater AB to social threat in comparison to control twins Non-ED co-twins had a AB towards social neutral stimuli	5
Kim et al., [58]	South Korea	AN = 31 HCs = 33	23.10 $\pm$ 9.35 22.18 $\pm$ 2.14	15.15 ± 2.51 20.91 $\pm$ 2.22	Dot-probe Task	Attentional bias towards happy vs. disgust vs. angry faces vs. neutral faces	Increased avoidance of angry faces in AN participants, increased attentional bias towards angry faces in HCs	9
Manuel and Wade [75]	Australia	AN = 24 HCs = 24	23.17 $\pm$ 10.52 23.25 $\pm$ 7.42	18.04 ± 2.48 22.84 $\pm$ 4.11	Emotional Stroop	Attentional bias towards angry vs. neutral faces	HCs displayed a AB towards angry faces whereas the AN group did not	7
Nuding et al., [77]	Germany	AN = 38 HCs = 36	15.48 $\pm$ 1.58 15.70 $\pm$ 1.76	16.19 ± 1.39 21.61 $\pm$ 4.07	Visual Scanning Task	Attentional bias assessed via eye-tracking with participants instructed to look at emotional stimuli, comprised of 80 photographs of five expressions (happy, angry, afraid, sad, neutral). The average percentage of dwell-time spent on the eye-area for each expression was measured	An exhibited increased dwell-time when viewing angry expressions compared to HCs. Observed AB was not restricted to anger but all negative and neutral expressions	6
Radix et al., [63]	Germany	AN = 32 HCs = 29	15.06 $\pm$ 1.29 16.00 $\pm$ 1.63	16.52 $\pm$ 1.62 22.02 $\pm$ 3.96	Dot-probe Task	Attentional bias following anxiety induction vs. low anxiety control task: Attentional biases towards underweight and overweight pictures (ED threat) vs. pictures of sculptures, and attentional biases towards happy and smiling faces (social Threat) vs. neutral faces	The anxiety induction did not affect the observed attention pattern. AN participants displayed an attentional bias towards underweight images, but no attentional bias was observed towards social stimuli regardless of valence	7
Schneier et al., [38]	USA	AN = 30 HCs = 74	26.9 $\pm$ 7.5 28.9 $\pm$ 7.6	*R* 16.0–18.5 N.S*	Dot-probe Task	Attentional bias towards angry vs. neutral faces	No attentional bias was found towards social threating faces for AN or HCs	7
Dipl-Psych et al., [49 ]	United Kingdom	AN = 49 HCs = 44	26.9 $\pm$ 7.8 25.8 $\pm$ 4.6	16.49 $\pm$ 1.27 21.61 $\pm$ 1.89	Dot-probe Task	Attentional bias towards social threat words vs. neutral words	No attentional bias was found towards social threat words for AN or HCs	8
Sfärlea et al., [64]	Germany	AN = 28 HCs = 24	15.37 $\pm$ 1.36 16.43 $\pm$ 1.56	16.41 $\pm$ 1.36 21.42 $\pm$ 3.26	Visual Scanning Task	Attentional bias was assessed via eye-tracking during a free-movement visual scanning task. Analysis included the initial orientation of attention and maintenance of attention measured by dwell time. A 2 × 2 stimulus array of underweight and overweight bodies and angry and happy faces was presented to participants in the experimental condition. In the control condition a 2 × 2 array of normal weight bodies and neutral faces were presented	Attentional bias was observed in both AN and HCs towards happy faces. Attentional avoidance was observed towards angry faces in AN but not HCs	7

AN, anorexia nervosa; AN-BP, anorexia nervosa, binge-purge subtype; AN-R, anorexia nervosa, restrictive subtype; HCs, healthy controls; HW-AN, healthy weight anorexia nervosa; MalAN, maltreatment anorexia nervosa; No MalAN, no maltreatment anorexia nervosa; RecAN, recovered anorexia nervosa; n/s, not specified; UW-AN, underweight anorexia nervosa

The most common source of bias across studies was not justifying sample sizes recruited, with only 8 studies (17.0%) conducting a priori power analysis. Further, 78.7% of studies did not use the same diagnostic tool to ascertain levels of AN psychopathology between AN and HCs. Moreover, 68% of studies did not report on education or IQ levels for group comparisons, or these parameters significantly differed between-groups, whilst 48.9% of studies did not report quantitative measures for state anxiety or depression. Four studies included AN participants who had a BMI ≥ 18.5, and the majority of studies did not report illness onset, illness duration, and average number of hospitalisations.

## Social attention

Attentional bias (AB) is described as the propensity to look for, and be attentive to, specific types of stimuli in the environment, often threats, whilst disregarding others [80].This component of selective attention has been termed engagement [80, 81]. Difficulties disengaging from specific types of information, have also been considered a form of AB [81, 82], alongside attentional avoidance occurring where attention is directed away from a perceived threat [82]. The outcome of our review identified 14 studies investigating AB in AN, within the context of social threat processing.

### Stroop paradigms

Five studies [33, 48, 57, 62, 75] employed modified Stroop paradigms [82, 83] to investigate AB towards social threat in AN patients with a focus on the engagement component of attention. In this paradigm, attentional bias is quantified as the latency to name the colour of emotional stimuli relative to neutral stimuli [83]. When increased latency is observed towards specific emotional stimuli this is inferred to be disease-relevant stimuli because it shows individuals are affected by the emotional content even though they are irrelevant to the colour-naming task [83].

Three studies [33, 48, 57] reported AB towards negative social stimuli in AN. One study [75] reported an AB towards social threat in HCs but not AN. One study found no evidence of an AB in a male AN sample [62].In a twin-study [48], twins with AN exhibited an AB towards social threat stimuli, whereas twins without AN exhibited an AB towards social neutral stimuli but not social threat.

### Dot probe task

Six studies [32, 37, 38, 49, 58, 63] employed the dot-probe task [84] to investigate AB in AN compared to HC samples**.** The dot-probe task has been used to measure engagement and disengagement components of attention, in addition to attentional avoidance [85]. In this task, participants are exposed to the simultaneous presentation of a threatening and neutral cue [85], replaced by a neutral probe appearing at the location occupied by either the threatening (congruent condition) or neutral cue (incongruent condition)[85]. Enhanced attentional engagement towards social threat is inferred when response latencies are shorter for congruent conditions than incongruent conditions [85]. Conversely, a deficit disengaging from social threat is inferred if response latencies are slower during incongruent conditions [85]. When response latencies are faster during incongruent conditions, this suggests attentional avoidance [85].

One study reported evidence of AB towards faces displaying rejection in AN, with specific difficulties in the engagement and disengagement component of attention observed [32]. Two further studies [37, 58], reported evidence of enhanced engagement towards social threat in AN in addition to an attentional avoidance of social threat [37, 58]. In one study [37], the discrepancy between enhanced engagement and attentional avoidance was shown to be associated with illness subtype, with AN-R participants exhibiting enhanced engagement and AN-BP displaying avoidance. In the other study [58], AN participants showed an attentional avoidance of social threat in the placebo arm of a trial testing the effects of oxytocin on attention, whereas the administration of oxytocin was shown to enhance attentional engagement towards rejection-cues. This finding showing attentional avoidance of social threat[58] contrasts with an earlier study which showed AB [32] towards this type of information.

Three studies reported no evidence of an AB for social threat information in AN using the Dot Probe Task [38, 49, 63]. Recruiting an adolescent sample[63], one study used the dot-probe task to compare, amongst other stimuli, angry versus neutral faces, following anxiety induction requiring participants to perform a difficult numerical task whilst receiving critical feedback. The anxiety induction was unable to modulate responsivity to social threat in either AN or HCs [63].

### Visual scanning paradigms

Visual scanning paradigms utilise eye-tracking technology to capture more direct measures of AB based on spatial (e.g., displacement) and temporal (e.g., velocity and acceleration) features of eye movements. An important distinction concerns the temporal dimension of the eye movement. Early attention reflects attentional orientation towards the emotional stimuli when first presented and has been used to indicate vigilance towards threat [86]. Late attention reflects the viewing pattern that occurs after the initial attentional orientation and is thought to reflect rumination or maintenance of attention towards the threatening stimuli [86].

Only two [64, 77] studies investigated scanning patterns towards social threat stimuli in adolescent AN samples. Firstly, it was found both AN and HCs exhibit a heightened attention on eye-regions that signal social threat, compared to eye-regions that signal acceptance but the propensity to dwell on these signals was greater in AN [77]. However, attention was not limited to social threat signals but neutral and negatively valanced social stimuli more broadly [77]. Conversely, in a paradigm where eye-movements were recorded whilst freely observing the simultaneous presentation of different images, including weight-based stimuli and angry and happy faces, both AN and HCs preferentially orientated their attention towards happy faces more than any other image category [64]. The overall attention for angry faces was significantly reduced in adolescent AN participants compared to HCs [64], reflecting lower prioritisation of attentional resources towards social threat in this group during ‘dual competition’[87] with weight-based stimuli.

### Affective priming task

One study used an affective priming task [88] to measure the automatic and unconscious mechanisms underlying AB in AN-R subtype [36]. This task presented schematic faces of positive, neutral, and negative valence for 100 ms, which is beyond the capacity for conscious awareness [88] followed by a positive or negative target word. A facilitation effect is inferred when response latencies are faster during congruent trials (i.e., prime and target word are matched by valence), and an inhibition effect when response latencies are slower in incongruent trials [36]. This study observed an amplified facilitation effect in AN-R subtype towards social threat compared to HCs [36], indicating aspects of their enhanced engagement difficulties towards this type of stimuli are underlined by automatic and unconscious processes. A greater inhibition effect towards social threat in AN-R was also observed [36].

## Social interpretation

Our social environments are constantly in flux and full of ambiguity, and the resolution of ambiguity is crucial in making sense of others’ behaviour [89]. Interpretation biases have been argued to represent automatic modes of inference.[89] (Table 2).

**Table 2:** Description of social interpretation experimental paradigms, study outcomes and risk of bias

Author and Date	Country	Participants	Age	BMI	Experimental Paradigm	Outcome measure	Main findings	Risk of Bias
			M SD	M SD				
An et al., [50]	South Korea	AN = 5 HCs = 51	*n/s* 23.43 $\pm$ 2.8	*n/s* 22.45 $\pm$ 4.86	Sentence Completion Task	Interpretation bias towards ambiguous social scenarios presented over audio. Participants listen to the sentences and write down as many short word completions to each scenario as they can, and then indicate with an Asterix the completion they endorse as the best scenario. Participants endorsed responses, in addition to the total sentence completions are rated as ‘benign, or ‘negative’ by five independent raters	Negative interpretation bias was observed in AN participants but not HCs	5
Cardi et al., [10]	United Kingdom	AN = 25 HCs = 30	26.7 $\pm$ 9.7 27.5 $\pm$ 3.5	21.3 $\pm$ 3.5 14.3 $\pm$ 1.7	Sentence Completion Task	Interpretation bias towards ambiguous social scenarios presented over audio. Participants initial responses and endorsed responses, in addition to the total sentence completions are rated as ‘positive’, ‘negative’ or ‘neutral’ by two independent raters	Negative interpretation bias was observed in AN participants but not HCs	7

AN, anorexia nervosa; AN-BP, anorexia nervosa, binge-purge subtype; AN-R, anorexia nervosa, restrictive subtype; HCs, healthy controls; HW-AN, healthy weight anorexia nervosa; MalAN, maltreatment anorexia nervosa; No MalAN, no maltreatment anorexia nervosa; RecAN, recovered anorexia nervosa; n/s, not specified; UW-AN, underweight anorexia nervosa

### Sentence completion tasks

Two studies [39, 50] investigated interpretation biases to social stimuli in AN by asking participants to provide as many endings as possible to stem sentences depicting socially ambiguous scenarios (e.g., ‘As you walk into a group of people, they stop talking because they were talking about…’). Interpretation biases are calculated as the percentage of negative, positive, and benign responses, as well as the valence of participants’ first and endorsed response (i.e., the one that was deemed the best completion. [39, 50] First responses were significantly more likely to be negative and significantly less likely to be positive or benign in AN participants compared to HCs.[39] Furthermore, percentage of negative response was significantly greater, and the percentage of positive and benign responses were significantly lower, in AN participants compared to HCs [39, 50].

## Social memory biases

Only three papers investigated social memory processes in relation to social rejection in AN [40, 51, 75] (Table 3).

**Table 3:** Description of social memory biases experimental paradigms, study outcomes and risk of bias

Author and Date	Country	Participants	Age	BMI	Experimental Paradigm	Outcome measure	Main findings	Risk of Bias
			M SD	M SD				
Jänsch et al., [40]	United Kingdom	AN = 28 HCs = 28	27.11 $\pm$ 7.51 28.21 $\pm$ 7.03	16.36 $\pm$ 1.31 23.50 $\pm$ 3.76	Emotional Memory Test	Sixty positive and negative self-referent words, matched for length, frequency, and meaningfulness, where presented to participants for 500 ms. Participants categorised these words as something they would like or dislike to be described as by pressing a labelled key on the keyboard. Participants were later asked to recall as many of the words as possible	AN participants recalled fewer words regardless of emotional valence vs. HCs. Within group differences revealed positive words were more likely to be recalled than negative words in AN	7
Manuel and Wade [75]	Australia	AN = 24 HCs = 24	23.17 $\pm$ 10.52 23.25 $\pm$ 7.42	18.04 ± 2.48 22.84 $\pm$ 4.11	Negative affective memory and recognition bias task	Sixty positive and negative self-referent words were shown to participants on a computer screen. After seeing all words, participants had 5-min to write down as many as they could remember, a measure of memory bias. At the end of the 5-min period, participants were shown 120 words (including the 60 shown previously) and asked to respond yes or no to whether they had seen the words previously, a measure of recognition bias	AN were significantly more likely to exhibit negative memory bias. There were no significant group differences in recognition bias. Negative memory bias was shown to mediate the relationship between AN symptoms and difficulties with emotional regulation	7
Via et al., [51]	Spain	AN = 20 HCs = 20	28.40 $\pm$ 9.30 28.15 $\pm$ 8.62	16.94 $\pm$ 1.26 20.99 $\pm$ 1.82	Critical Feedback Paradigm	Participants photographs were taken by the experimenter. Fifty-four pictures of individual’s face were presented to participants on a screen during fMRI scanning. Participants were provided with pre-programmed feedback on whether each individual accepted, rejected or provided no response, on an offer to meet the participant after viewing their photo. After the experiment participants were shown seventy faces and asked to recall whether they had seen the individuals before	No between-group differences were observed in overall recall rates for positive, neutral, or negative feedback between AN and HCs. There was a general tendency to recall rejecting feedback in both groups	8

AN, anorexia nervosa; AN-BP, anorexia nervosa, binge-purge subtype; AN-R, anorexia nervosa, restrictive subtype; HCs, healthy controls; HW-AN, healthy weight anorexia nervosa; MalAN, malnourished anorexia nervosa; No MalAN, no malnourishment anorexia nervosa; RecAN, recovered anorexia nervosa; n/s, not specified; UW-AN, underweight anorexia nervosa

### Emotional memory test

Two studies [40, 75] utilised an emotional memory test to investigate involuntary recollection of socially relevant memories. In the first study [40], participants were presented with words describing positive and negative personality states, and no social element. After a suitable consolidation period, participants were asked to recall as many words as possible. Compared to HCs, AN patients recalled less positive and negative personality traits in the context of intact memory for non-social words. Within-group analysis further revealed AN patients were more likely to recall positive than negative words. When controlling for the effects of depression, the difference between AN and HCs was less pronounced [40]. These findings contrasted with a second study [75] that observed a social memory bias towards negative streams of social information in AN with this group more likely to recall negative personality states compared to positive traits.

### Critical feedback paradigm

One study [51] used a critical feedback paradigm to study the relationship between being the recipient social judgment and social memory biases. On the first day, participants were told they would be part of a multi-centre study to investigate first impressions. Participants were shown 70 faces of other individuals, each displaying a neutral expression. Participants were tasked with indicating whether they would accept or reject a future opportunity to meet them, as well as the degree they would like to meet them. Participants were also photographed and told they would be rated similarly by the other individuals. However, these individuals were fictitious, and faces were sourced from a database. On the second day, participants were told whether the fictitious individuals had accepted or rejected to meet them or did not respond. Subsequently, participants were presented with images of individuals’ faces once more and asked to recall if they had been seen previously and what type of feedback the “individual” had given. Results indicated that both AN and HCs were able to recognise with high accuracy the faces presented, and this was not influenced by the type of social feedback received. Both groups more accurately remembered receiving rejecting feedback, in comparison to being accepted or receiving no feedback.

## Emotional recognition and emotional regulation

### Emotion recognition tasks

Thirteen studies used an emotion recognition task to investigate AN participants’ ability to identify basic threatening emotions, including anger and disgust which have been shown to operate as social signals of rejection [90, 91], in faces [40, 42–44, 55, 59–61, 70, 71, 74, 79], voices [43], and body movement [56] (Table 4).

**Table 4:** Description of emotional recognition and emotional regulation experimental paradigms, study outcomes and risk of bias

Author and Date	Country	Participants	Age	BMI	Experimental Paradigm	Outcome measure	Main findings	Risk of Bias
			M SD	M SD				
Ambwani et al., [69]	United Kingdom	AN = 33 HCs = 37	N/S	N/S	Critical Feedback Videos	Video-clips of job supervisors providing critical feedback (varying in degrees of dominance and submissiveness and coldness and warmth)	AN patients perceived more coldness overall and where more likely to endorse responding to feedback in a cold manner, compared to HCs	5
Dapelo et al., [59]	United Kingdom	AN = 35 HCs = 42 (AN-R = 17; AN-BP = 18)	27.54 $\pm$ 8.36 26.98 $\pm$ 7.55	15.33 $\pm$ 1.74 22.53 $\pm$ 2.63	Facial Emotional Recognition task	Black and white images of blended emotions; happiness, fear, sadness, disgust, and anger were displayed. Participants are asked to identify the emotions with accuracy recorded	AN patients were less accurate recognising expressions of disgust when it was at 90% proportion. They also displayed a significantly higher preference to interpret non-angry faces as anger compared to HCs	7
Gramaglia et al., [60]	Italy	AN = 39 HCs = 48 (AN – R = 27; AN-BP = 12)	30.59 $\pm$ 3.00 33.19 $\pm$ 3.37	16.3 $\pm$ *n/s* 21.82 $\pm$ *n/s*	Facial Emotion Recognition Task	Black and white images of the six basic emotions (anger, disgust, fear, happiness, sadness, surprise), and neutral faces. Participants are asked to identify the emotions with accuracy recorded	AN patients were significantly more likely to correctly identify disgust compared with HCs	6
Jänsch et al., [40]	United Kingdom	AN = 28 HCs = 28	27.11 $\pm$ 7.51 28.21 $\pm$ 7.03	16.36 $\pm$ 1.31 23.50 $\pm$ 3.76	Facial Emotion Recognition Task	Images of the six basic emotions (anger, disgust, fear, happiness, sadness, surprise), morphed with neutral faces from 0% emotion (neutral) to 100% emotion (full emotion) were presented in 10% increments. Total scores were calculated for each emotion separately, for accuracy, reaction times, and misclassification	AN participants compared to HCs, were less accurate at identifying emotions, responded more slowly, and misclassified more faces. No particular emotion(s) were identified more accurately, more quickly, or misclassified more often	7
Kessler et al., [42]	Germany	AN = 31 HCs = 48	22.9 $\pm$ 7.7 22.8 $\pm$ 5.8	16.3 $\pm$ 1.9 *n/s*	Facial Emotional Recognition Task	Images of the six basic emotions (anger, disgust, fear, happiness, sadness, surprise). Participants are asked to identify the emotions with accuracy recorded	No significant differences in emotional recognition between AN and HCs	7
Kucharska-Pietura et al., [43]	Poland	AN = 30 HCs = 30	20.2 $\pm$ 4.4 25.2 $\pm$ 4.0	15.2 $\pm$ 1.7 *n/s*	Facia and Vocal l Emotional Recognition Task	Images of 36 emotional faces depicting nine emotions (interest, happiness, surprise, sadness, disgust, contempt, anger, shame and fear) were presented to participants. Participants also listened to a set of 35 sentences, with the speaker’s tone of voice reflecting one of the six basic emotions (happiness, sadness, fear, anger, surprise, disgust) or neutral in valence. Participants are asked to identify the emotions with accuracy recorded	AN participants were significantly poorer at recognising negative emotions in faces, and both positive and negative emotions in voices	7
Mendlewicz et al., [44]	Belgium	AN = 36 HCs = 32 (AN-R = 20; AN-BP = 16)	19.5 ± 3.8 21.2 ± 2.1	15.4 $\pm$ 2.1 20.4 $\pm$ 2.3	Facial Emotional Recognition Task	Images of 40 emotional faces (happiness, anger, sadness, disgust, fear) that varied in intensity from 0% (neutral), to 100% full emotion. Participants were asked to correctly identify the emotions across the varying intensities	No significant differences between AN and HCs in emotional recognition. AN showed recognition advantages for happy, sad and afraid expressions	8
Pollatos et al., [55]	Germany	AN = 12 HCs = *n/s*	23.86 ± 4.25 22.39 ± 4.78	16.34 $\pm$ 1.14 22.95 $\pm$ 4.52	Facial Emotional Recognition Task	Participants were presented with 240 images of faces depicting neutral, sad, happy, fearful, angry, and disgusted expressions. Participants are asked to identify the emotions with accuracy recorded	AN participants made more mistakes in emotional recognition, for neutral, sad and disgusted expressions, compared to HCs	8
Sfärlea et al., [70]	Germany	AN = 26 HCs = 37	15.2 ± 1.7 15.2 ± 1.7	15.4 $\pm$ 1.2 20.1 $\pm$ 2.4	Facial emotional recognition task	Images of 320 faces depicting neutral, happy, sad, afraid, and angry expressions. Participants are asked to identify the emotions with accuracy recorded	No significant differences between AN and HCs in emotional recognition for anger	7
Wyssen et al., [71]	Germany/Switzerland	AN = 61 HC = 130	22.87 ± 4.57 21.53 ± 2.18	17.05 $\pm$ 1.63 22.01 $\pm$ 2.63	Facial emotional recognition task	Participants were presented with six basic emotion expressions (fear, anger, disgust, happiness, sadness, surprise), plus a neutral expression and asked to identify the emotions displayed with accuracy recorded	No significant differences were observed between those experiencing AN and HC	6

AN, anorexia nervosa; AN-BP, anorexia nervosa, binge-purge subtype; AN-R, anorexia nervosa, restrictive subtype; HCs, healthy controls; HW-AN, healthy weight anorexia nervosa; MalAN, malnourished anorexia nervosa; No MalAN, no malnourishment anorexia nervosa; RecAN, recovered anorexia nervosa; n/s, not specified; UW-AN, underweight anorexia nervosa

Only 3 studies [55, 59, 60] reported different processing of social threat emotions in AN patients'compared to HCs. Two studies [55, 59] revealed the ability to recognise disgust expressions in AN was significantly diminished [55, 59]. Conversely, 1 study [60] reported AN patients have enhanced abilities to process disgust. The rest of studies either reported on difficulties in emotional recognition [40, 43, 56, 61, 79], rather than a difference specific to social threat, or no evidence of emotional recognition impairments [42, 44, 70, 71], in AN participants.

### Interpersonal efficacy task

One study [69] investigated the relationship between social threat processing and interpersonal self-efficacy, namely one’s ability to engage in a variety of interpersonal behaviours to effectively manage and regulate emotions. Participants were presented with mock positive and critical ‘feedback’ from job supervisors. Those with AN but not HCs displayed a negative interpretation bias perceiving more coldness from their feedback. AN participants also tended to endorse responding in a cold manner to both positive and critical feedback. This highlights an association between misinterpreting social cues and misalignments in social reciprocity, which we speculate may lead to a barrier for effective co-regulation.

## Affective, physiological, and behavioural response to social rejection and interpersonal stress

We found 15 papers investigating emotional, behavioural and physiological response to social threat and rejection in AN (Table 5).

**Table 5:** Description of affective, physiological, and behavioural response experimental paradigms, study outcomes and risk of bias

Author and Date	Country	Participants	Age	BMI	Experimental Paradigm	Outcome measure	Main findings	Risk of Bias
Crucianelli et al., [65]	United Kingdom	AN = 20 HCs = 25	24.0 $\pm$ 12.75 26.0 $\pm$ 7.25	14.38 $\pm$ 1.68 21.03 $\pm$ 3.04	Affective Touch Paradigm	Participants rated the pleasantness of slow stroking touches on the forearm known to activate the CT afferent system vs. fast stroking touches on the forearm not associated with CT afferent activity. Whilst rating touches participants were simultaneously shown accepting, rejection and neutral faces	Social rejection faces did not modulate the perception of pleasantness of affective touch in both groups	7
Cartaud et al., [73]	France	AN = 29 HCs = 30	27.15 $\pm$ 9.59 24.79 $\pm$ 6.27	18.61 $\pm$ 3.6 22.35 $\pm$ 3.45	Interpersonal Distance Judgement Task	Participants observed virtual characters displaying different facial expressions and selected the interpersonal distance they felt comfortable with the character with electrodermal activity recorded	AN participants and HCs both preferred increased interpersonal distance for characters with angry faces. AN participants compared to HCs displayed blunted electrodermal activity	4
Het et al.,[41 ]	Germany	AN = 18 HCs = 26	*N/S*	*N/S*	Trier Social Stress Test	Physiological responsivity to interpersonal stress	AN patients exhibited a blunted cortisol response and reduced salivary alpha-amylase levels compared to HCs	6
Meneguzzo et al., [52]	Italy	AN = 32 HCs = 34	22.81 $\pm$ 6.94 24.21 $\pm 2.57$	17.03 $\pm$ 1.19 21.78 $\pm$ 3.41	Cyberball Paradigm	Affective responsivity to inclusion vs. exclusion	AN participants compared to HCs reported lower meaningful existence and reduced self-esteem on the need threat scale. Dependence/incompetence, negativity/pessimism, and self-sacrifice were significant predictors of scores on the NTS following exclusion	5
Meneguzzo et al., [66]	Italy	AN = 42 HCs = 50	24.62 $\pm$ 8.26 24.16 $\pm 3.41$	16.68 $\pm$ 0.87 21.45 $\pm$ 2.96	Cyberball Paradigm	Affective and behavioural responsivity to inclusion vs. exclusion	After exclusion, AN compared to HCs reported significantly higher thoughts about restricting eating in addition to lower meaningful existence and reduced self-esteem on the need threat scale	6
Miller et al., [67]	United States	AN = 25 HCs = 73	15.6 $\pm$ 1.9 16.0 $\pm 1.23$	16.1 $\pm$ 14.9 $n/s$	Stress Induced Speech Task (SIST)	Self-report positive and negative valence and heartrate responsivity to interpersonal stress induced by speaking about a traumatic event	AN patients displayed a muted pulse rate compared to HCS during the stress task. AN patients compared to HCs displayed higher negative affect throughout the SIST	6
Miller et al., [35]	United States	RecAN = 17 HCs = 40	15.53 $\pm$ 1.42 16.16 $\pm 0.88$	17.90 $\pm$ 15.57 $21.55$ $\pm$ 24.66	Stress Induced Speech Task (SIST)	Self-report positive and negative valence and heartrate responsivity to interpersonal stress induced by speaking about a traumatic event	RecAN participants exhibited higher levels of negative affect during the TSST compared to HCs. RecAN participants did not exhibit a muted heart rate response during the TSST	4
Monteleone et al., [53]	Italy	AN = 15 HCs = 8	20.2 $\pm$ 2.2 $23.6\pm$ 2.2	16.3 $\pm$ 1.2 21.1 $\pm$ 2.4	Trier Social Stress Test	Saliva cortisol and saliva α-amylase responsivity to interpersonal stress	AN patients exhibited a higher cortisol response and blunted α-amylase response compared to HCs	7
Monteleone et al., [26]	Italy	MalAN = 12 No MalAN = 12 HCs = 17	24.0 $\pm$ 6.6 23.3 $\pm$ 5.2 26.0 $\pm$ 2.5	16.8 $\pm$ 1.3 16.8 $\pm$ 1.5	Trier Social Stress Test	Self-report anxiety and saliva cortisol responsivity to interpersonal stress	AN patients exhibited a blunted cortisol response compared to HCs. MalAN exhibited a reduced anxiety increase after TSST compared to No MalAN and HCs	6
Monteleone et al., [54]	Italy	AN = 21 HCs = 27	24.06 $\pm$ 4.10 $25.10\pm$ 3.25	17.10 $\pm$ 2.02 22.13 $\pm$ 2.52	Trier Social Stress Test	Self-report anxiety, hunger perception and amount of desired food in response to interpersonal stress	AN patients exhibited higher anxiety scores compared to HCs. Hunger perception and desire for food significantly decreased in AN relative to HCs	6
Schmalbach et al., [45]	Germany	HW-AN = 26 UW-AN = 8 HCs = 26	26.50 $\pm$ 6.11 25.13 $\pm$ 4.79 25.00 $\pm$ 5.50	20.7 $\pm$ 3.00 16.0 $\pm$ 1.00 23.08 $\pm$ 3.30	Trier Social Stress Test	Self-reported cognitive appraisal during debrief before the TSST. Self-reported stress levels, and salivary cortisol responsivity to interpersonal stress after the TSST	AN patients compared to HCs exhibited higher levels of cognitive appraisal related to threat, and reduced self-concept of one’s own abilities AN patients displayed delayed and blunted cortisol reactivity compared to HCs, Stressed increased from pre to post TSST on the VAS but was comparable between groups	5
Schmalbach et al.[46]	Germany	AN = 19 HCs = 19	26.05 $\pm$ 5.49 24.21 $\pm$ 5.54	18.70 $\pm$ 3.30 24.23 $\pm$ 3.04	Trier Social Stress Test	Self-reported cognitive appraisal during debrief before the TSST. Heartrate response following the TSST	AN patients compared to HCs exhibited higher levels of cognitive appraisal related to stress and threat, and reduced self-concept of one’s own abilities AN patients demonstrated a blunted HR response compared to HCs	5
Schmalbach et al., [47]	Germany	AN = 19 HCs = 19	25.26 $\pm$ 5.53 24.16 $\pm$ 5.12	17.80 $\pm$ 1.70 22.12 $\pm$ 1.32	Trier Social Stress Test	Chewing frequency and food intake during a test meal following interpersonal stress	AN patients exhibited reduced chewing frequency and indigestion compared to HCs following the TSST Within-group analysis revealed chewing frequency or ingestion did not differ in AN pre and post TSST Following TSST AN patients increased their drinking volume	5
Vaz-Leal et al., [78]	Spain	AN = 15 HCs = 22	22.0 $\pm$ 3.6 21.7 $\pm$ 2.3	16.7 $\pm$ 0.8 21.6 $\pm$ 1.1	Trier Social Stress Test	Saliva cortisol levels after dexamethasone suppression test (DST), following the TSST	AN patients exhibited a weaker capability to suppress cortisol following DST compared to HCs AN patients displayed a profile of cortisol liberation throughput the TSST very similar to HCs	7
Zonnevylle-Bender et al., [68]	Netherlands	AN = 10 HCs = 22	15.5 $\pm$ 1.8 $n/s$	16.2 $\pm$ 1.2 $n/s$	Trier Social Stress Test	Self-reported emotional arousal, heartrate responsivity and saliva cortisol levels following interpersonal stress	AN patients reported higher tension, exhibited blunted cortisol response and lower HR compared to HCs, following TSST	7

AN, anorexia nervosa; AN-BP, anorexia nervosa, binge-purge subtype; AN-R, anorexia nervosa, restrictive subtype; HCs, healthy controls; HW-AN, healthy weight anorexia nervosa; MalAN, malnourished anorexia nervosa; No MalAN, no malnourishment anorexia nervosa; RecAN, recovered anorexia nervosa; n/s, not specified; UW-AN, underweight anorexia nervosa

### Affective touch paradigm

One study [65] examined the social modulation of touch pleasantness by using gentle brush whilst concomitantly presenting faces depicting acceptance, rejection, or neutral expressivity. The authors found evidence of reduced pleasantness ratings (i.e., tactile anhedonia) during tactile optimal touch in AN but not HCs. Exposure to rejection faces did not modulate the experience of pleasantness of touch in AN and HCs [65].

### Stress induced speech task

One study [67] utilised a stress induced speech task, requiring participants to first describe the most traumatic experience they encountered (stress task), and then engage in a ‘free association task’ (control task) where participants could discuss any topic of their choosing [92]. Both components are audiotaped, which is assumed to increase the degree of interpersonal stress experienced [92]. Self-reported negative affect was higher in AN participants compared to HCs, before and during both speech tasks. Furthermore, although heartrate was significantly lower in AN participants compared to HCs across all timepoints and conditions, there was a noticeable spike in heartrate in AN participants 5-min into the stress task. This initial spike was accompanied by a noticeable dip in heartrate 10-min into the stress task in AN participants’, but this phenomenon was not present in HCs whose heartrates remained steady [67].

### Trier social stress test

The Trier Social Stress Test (TSST) [93] is considered the gold standard [94] experimental paradigm for evaluating the neurobiological response to acute interpersonal stress in humans. The TSST requires the participant to speak in front of an unresponsive audience and complete a surprise challenging mental arithmetic task [93]. This exposes participants to thoughts of being socially judged and uncontrollability and has been shown to be highly anxiety provoking [94].

Nine studies [41, 45–47, 53, 54, 68, 76, 78] utilised the TSST to investigate SRS in AN. Before, during, and after the TSST, participants completed questionnaires assessing anxiety and mood [54, 68], physiological measures of heartrate responsivity [46, 68] and neurophysiological measures of Hypothalamic–Pituitary–Adrenal Axis (HPA Axis) responsivity (i.e., saliva cortisol concentrations [45, 68] and autonomic nervous system activity (i.e., saliva α-amylase concentrations [53]). Five studies [45, 46, 54, 68, 76] reporting on affective reactivity found adolescents [68] and adults [45, 46, 54, 76] with AN are more emotionally sensitive to experiences of social-evaluative threat than HCs. One study[54] reported a significant proportion of AN participants opted to drop-out after learning what the TSST entails at the start of the experiment. Two studies [54, 76] found the magnitude of anxiety across all experimental time periods was higher in AN participants compared to HCs, peaking in response to the TSST. A further study [68] observed a similar pattern with higher overall levels of self-reported levels of tension peaking after interpersonal stress. Two studies [45, 46] showed increased stress, with an additional study [54] reporting higher body dissatisfaction, in AN after exposure to the TSST. Body dissatisfaction was associated with overall anxiety levels and the anxiety experienced during the TSST [54]. Further comparisons [76] between AN participants with and without childhood trauma revealed a smaller increase in anxiety in the former group.

Analysis on physiological reactivity found that in comparison to HCs, saliva α-amylase concentrations were significantly reduced in AN immediately before [53] and after TSST exposure [41, 53]. Similarly, heartrate variability was observed to be significantly lower in AN patients but not HCs, during the TSST [46, 68].

Six studies [41, 45, 53, 68, 76, 78] reported on cortisol concentrations with inconsistent findings. The majority of studies found reduced levels in adolescent [68] and adult [41, 45, 78] patients relative to HCs in response to the TSST. One study reported the inverse, observing higher baseline and post-TSST cortisol levels in AN [53]. Further findings [45, 76] showed when the total amount of cortisol released was compared, AN and HCs exhibited a similar pattern. However, the amount of cortisol released relative to baseline differed [41, 45], with significant reductions observed in AN participants [41, 45]. These reductions were further observed to be more pronounced in an patients with childhood trauma compared with those without [76]. This indicates a greater level of desensitisation of the stress response in the former group. Whilst one study [78] found that both AN and HCs exhibited a similar profile of cortisol liberation during the TSST, another study [45] observed a delayed onset in AN.

Two studies [47, 54] explored eating behaviours following the TSST. The first study [47] observed increased chewing frequency during a test meal in both AN and HCs. Increased chewing frequency has been linked to reduced hunger [95]. However, whilst food intake was reduced in both groups, only HCs were preferentially impacted by the task. This may be because AN patients generally exhibit low food intake. The latter study [54] found self-reported hunger and desire for food significantly decreased, and this negatively correlated with state levels of anxiety and the anxiety felt during the TSST.

### Interpersonal distance judgement task

One study [73] investigated interpersonal distance in AN, focusing on how other’s facial expressions moderate proxemics. Participants were exposed to virtual characters depicting angry, neutral or happy expressions who were located either near or far the participants’ personal space. Participants were required to select the interpersonal distance at which they could comfortably interact with the character, while electrodermal activity was recorded. Results showed AN and HCs responded similarly, both preferring larger interpersonal distances for angry characters, compared with neutral or happy characters. However, the electrodermal responses was blunted in the AN sample. Both groups rated the valence of angry characters less positively and more arousing than neutral and happy characters.

### Cyberball paradigm

Two studies [52, 66] used the Cyberball [96] task to investigate the effects of experiencing experimentally induced ostracism in AN. Cyberball is a virtual ball tossing game, where participants are led to believe they are playing with other players, but they are in fact playing with a preprogrammed algorithm set to either include or exclude the participant from the game [96]. Inclusion is achieved through increasing the amount of ball tosses the participant receives to at least an equal amount to the other players, whilst ostracism is achieved through significantly reducing the amount of ball tosses the participant receives, so they in effect become excluded and passive observers of the game [96]. Overall, AN participants estimated that they received a significantly reduced number of ball tosses, indicating a heightened perceptual awareness of being ostracised [52, 66]. AN participants also reported greater negative affect compared to HCs following being ostracised [52]. Moreover, although post-ostracism both AN participants and HCs exhibited a depletion in four fundamental psychological needs associated with social connection [52, 66], encompassing belonging, self-esteem, meaningful existence, and control [97], this depletion was observed to be enhanced in AN [52, 66], and especially in the domain of self-esteem and meaningful existence [52, 66]. Impoverishment of these needs is postulated to be a contributory factor in the distressing phenomenology of ostracism [97], further demonstrating evidence of a more intense emotional response to rejection-relevant cues in AN [52, 66]. Further analysis revealed, despite the fact self-reported thoughts about restricting eating remained higher in AN compared to HCs, exposure to ostracism did not moderate this parameter [66].

## SRS as a trait or state factor

Seven studies [31–33, 35, 37, 46, 58] attempted to investigate whether the cognitive mechanisms underlying SRS become dysfunctional because of the effects of starvation and malnutrition or can be considered trait features of AN. One study utilised the Stroop [33] and found AN and RecAN participants both displayed AB towards social threat information. Two studies [31, 32] utilising the dot-probe task with RecAN groups reported contrasting results. The first study [32] found an AB towards social threat in RecAN, whereas the latter study [31] found no evidence of an AB towards social threat in RecAN. One study [35] observed adolescent RecAN participants experience heightened distress and negative emotions in response to the Stress Induced Speech Task compared to HCs, but there was no muted heart rate response. This contrasted with findings from a further study that observed reduced heart rate in adult weight-restored patients [46].

## Discussion

The primary goal of the current review was to establish whether individuals diagnosed with AN respond differentially and more sensitively to exposure to social threat or rejection-based stimuli. Our main findings showed the current evidence base on SRS in AN can be considered of good quality, with many studies showing SRS in AN, in line with previous findings in general ED groups [26].

In the attention literature, there was consistent evidence showing alterations in the way AN patients engage with social threat information. Most studies showed enhanced engagement towards social threat in AN [32, 33, 36, 37, 48, 57, 77] with a smaller subset also observing delayed disengagement [32, 36, 77]. One study [36] showed enhanced engagement and delayed disengagement were driven by both automatic and unconscious processes. Results on attentional avoidance were inconclusive with only 3 studies [37, 58, 64] reporting on this process. This is an area that warrants further investigation as attentional avoidance could be used to reduce arousal triggered by social threat [98] and may therefore play a key maintenance role. Overall, the observed AB towards negative streams of social information in AN has clinical relevance because it suggests that the social world of patients may appear more hostile, thus corroborating some patients’ narratives [10, 99] while also identifying the underlying mechanisms.

It is important to note that not all studies found altered attention towards social threat in AN. This includes 2/5 studies [62, 75] utilising the Stroop and 3/6 [38, 49, 63] studies utilising the dot-probe. The absence of AB in the studies using the Stroop may be attributed to methodological and clinical, heterogeneity. In one study [75], AB towards social threat was observed in HCs, which is an anomalous finding to comparable studies [33, 48, 57]. The other study [62] included an exclusively male sample, which could have accounted for lack of findings given that risk factors for AN may vary across gender [100]. The absence of AB in the three studies utilising the dot-probe is more difficult to interpret. One study found no evidence of AB in an adolescent sample following an interpersonal stress task [63]. It is possible AB becomes more detectable as the illness progresses, or the stress task interfered with AB detectability. Absence of AB was also observed in two studies using rejection-based faces [38, 63] and one study using threat-based words [49]. This is in contrast with what found by other comparable studies. While methodological and clinical heterogeneity may account for the observed differences, it has also been suggested that the dot-probe task is associated with problems of reliability [101] which speaks for the need for future research to strengthen the evidence on aberrant social attention mechanisms in AN.

In other cognitive domains of SRS there was ubiquitous evidence for negative interpretation biases across a small number of studies [39, 50, 69], showing patients with AN are more prone to seeing rejection in ambiguous social scenarios [39, 50], with a further study [69] AN patients are more likely to perceive less warmth in others. Negative interpretation biases could have important clinical consequences, for example some AN patients discuss a reluctance to disclose their difficulties to peers and loved ones because of these cognitions [20], which may lead to a barrier in accessing emotional support [102]. Mirroring this, some caregivers and professionals also discuss feeling worried about what to say or do [20], because their well-meaning interactions may exacerbate patients’ distress who incorrectly interpret these actions as signalling a lack of affection and rejection [102].

The relationship between memory biases and AN was less clear due to only three studies [40, 51, 75] investigating this domain in relation to social threat. Results were inconsistent, with only one of these studies [75] reporting a specific recall bias for negative personality traits. Expanding on this, all studies sourced investigated semantic components of memory [103] but memory biases are complex, subserved by multiple mechanisms [103]. We found an absence of studies investigating episodic memories of previous social rejection experiences and given that SRS may arise from past experiences of rejection [6] this represents a significant gap in our knowledge that should be addressed in future research.

Inconsistent findings in relation to emotional recognition abilities in AN corroborated a prior finding [4]. Only 2 studies[55, 59] reported reduced capacity to recognise disgust, a potential signal of rejection [90]**.** One study [60] showed disgust recognition was enhanced. Five studies [40, 43, 56, 61, 79] reported emotional recognition deficits that were generalised across emotions, whereas four studies [42, 44, 70, 71] found emotional recognition abilities in AN comparable to HCs. If a subset of patients with AN experience difficulty in emotional recognition, this casts doubt on the interpretation of 11 AB [31–33, 36, 38, 48, 57, 58, 62–64] studies, that each used facial expression stimuli. AB towards rejection cues could be adaptive with poor emotional recognition, as an alternative strategy to obtain information about possible threats in the social environment [104]. Alternatively, heightened vigilance towards social threat may interfere with the capacity for emotional recognition [104]. More research is needed to explore these hypotheses to identify if and how alterations to emotional recognition are associated with SRS in AN.

Patients with AN consistently react with heightened negative affect to social threat cues [45, 46, 54, 67, 68, 76] and experiences of rejection [52]. Interestingly an affective-physiological mismatch during social threat was also observed [41, 46, 53, 67, 78] characterised by an incongruence between heightened affect and blunted physiological reactions. A further study [35] showed physiological blunting, but not affective reactivity, remediates throughout the process of weight-restoration. The notion that bottom-up physiological signals influence top-down affect is widely accepted [105] and these observations allude to a possible mechanism for starvation having anxiolytic effects through physiological blunting. Exerting control over one’s internal milieu through restrictive eating is a concept that has recently been proposed [106], and theories proposing AN in-part functions to intentionally but unconsciously shunt affective reactivity are well established [11, 12, 107]. These preliminary findings prompt us to consider whether a domain-general perspective on affective shunting should be reconsidered in favour of an alternative hypothesis that postulates only physiological streams are inhibited. We also cautiously ponder whether being a healthy weight may incur additional costs through somatising interpersonal distress. This highlights the need to address our questions, as training that targets these mechanisms might be useful in treating AN and preventing relapse [105, 106, 108, 109].

Findings also support theoretical models that postulate SRS develops due to biological factors that increase vulnerability to developing certain socioemotional processing styles, in combination with adverse early experiences that activate them [6]. By comparing pairs of affected and unaffected twins of AN patients, it was found only affected twins displayed an AB to social threat, whereas unaffected twins exhibited a bias to social stimuli more broadly [48]. Whilst early adverse experiences were shown to be associated with AB to social threat [32]. These results imply genetics may influence social processing in AN, with adverse early experiences directing this predisposition towards enhanced engagement and delayed disengagement of social threat stimuli. More studies are needed to confirm this link, and determine the applicability of this model to other domains of SRS cognitive processing in AN.

A secondary objective of our review aimed to explore whether SRS represents a state or trait feature of the illness. Studies that speak to this question have explored relevant phenomena in RecAN groups. Four studies investigated RecAN groups [31–33, 35]; three found persistent difficulties in recovered individuals in the attentional [32, 33] and affective reactivity [35] domains. While such studies speak towards SRS being state independent, it is still possible that such observations are scars from the illness and not inherited traits. Longitudinal studies would bring clarity on the matter. Regardless, such findings raise concerns pertaining to the way in which SRS may impact psychosocial wellbeing and functioning in recovered groups. The challenge for future research will be to clarify the extent SRS may impact recovery and relapse.

Overall, our results begin to elucidate some of the mechanisms that may underpin SRS in AN, supporting theoretical suggestions that restrictive eating may function as a maladaptive coping mechanism to patient’s perceptions of a hostile social world. Interpreted within frameworks of current disease models [11, 12, 106, 110] the pursuit of thinness may serve dual regulatory functions; first as an anticipatory mechanism to establish agency over one’s physical presentation to satisfy the underlying need to belong [12, 110] and remedy rejection fears, and second, as an emotion regulation strategy [11, 12, 106] by attenuating the intensity of rejection-related, physiological distress [106]. This may elucidate why patients attribute value to illness-maintain factors by persisting in weight-loss behaviours [13] despite the harmful effects to health and wellbeing [111, 112].

Our findings may have clinical relevance, particularly in the realm of cognitive bias modification training which has demonstrated utility in targeting mechanisms underlying SRS [89]. Correspondingly, an emerging literature shows AB [20, 21], and interpretation biases [20–22] can be modified in AN towards neutral patterns of social cognitive processing. Our findings also underscore the potential benefit integrating social support into treatment protocols, to enhance patients’ interpersonal experiences and fulfil their belonging needs, which may lessen the drive for thinness.

## Limitations and future research recommendations

There are several limitations to our review. Firstly, due to the lack of studies that investigated male samples we were unable to adequately gauge whether SRS is a relevant feature of this clinical group. Thus, our main findings pertain to females only. The one study [62] that found no AB in an all-male AN sample, may reflect gender-differences in social threat processing. The broader literature on rejection suggests being female amplifies the risk of SRS [6] because of learnt gender roles and heightened exposure to interpersonal stress, in childhood and adolescence [6, 113]. These differences may also contribute to the higher prevalence of AN among females [114]. Future research should focus on including adequate samples of both males and females with AN, to comprehensively assess gender effects.

Secondly, we only sourced emotional recognition tasks that incorporated social threat images. This may have neglected other aspects of social cognition, such as empathy [7] and perspective-taking [115], that may play a role in SRS [7, 115].

Although we sourced studies relationship between SRS and clinical symptoms, as outlined in our preregistration protocol, there was insufficient data to conduct meaningful analysis. Future research should explore the role of SRS in AN, regarding depression, anxiety and other related symptoms, to provide a deeper understanding of potential interactions.

We also did not fully investigate differences in social threat processing between adolescence and adult samples. Adolescence is an important period in cognitive development and a time where social relationships form a critical basis for one’s identity [116]. It is also a period marked by hypersensitivity to rejection [6, 116]. It is reasonable to conclude that SRS will manifest across all ages in people with AN. However, we found limited studies investigating adolescent samples. What remains unclear is whether the underlying mechanisms of SRS are consistent across different age groups, or if they evolve as the illness progresses. More research is needed on adolescent samples, subclinical populations, and the effects of illness duration, as this will better characterise the stability and enduring nature of SRS in AN.

## Conclusion

This study supports SRS as a key feature of AN. Patients with AN compared to HCs, were generally more likely to expect rejection, readily perceive rejection and react with heightened affect to social threat and rejection cues, with limited evidence suggesting this persists in recovery. Physiological reactivity was shown to be blunted, with one study showing this remediates through weight-gain. Clinicians should be mindful of SRS in their patients, which could influence therapeutic alliance, and treatment outcomes. More robust research is needed to determine the efficacy of interventions for SRS in AN, and mechanistic pathways from SRS to development of symptoms in this group.

## Supplementary Information

Additional file 1.

## Data Availability

No datasets were generated or analysed during the current study.
