# Supplementary material for: Social rejection sensitivity and its role in anorexia nervosa: a systematic review of experimental literature
Source: J Eat Disord. 2025 Jul 10;13:134. doi: 10.1186/s40337-025-01261-7 (PMC12247325; doi:10.1186/s40337-025-01261-7)
Supplement: Supplementary file 1 — Additional file 1. [file 40337_2025_1261_MOESM1_ESM.docx]

Risk of Bias Assessment of the studies included in the systematic review assessed using an adapted version of the Newcastle Ottawa Scale (case-control study). The answers selected in response to each of the seven questions included from the scale are reported here, with an overall quality score reported for each study included in the outcome tables of our results, which has been incorporated into the main text.

This questionnaire contains measures bias across three outcomes: selection, comparability, and exposure. Selection contains four questions, that can be answered by selecting one of two to three options, with only option *a* scoring for low risk of bias. Comparability contains one question, that can be answered with two options, with both options (*a & b*) scoring for low risk of bias. Exposure contains two questions, with question one containing five options, with options *a & b* scoring for low risk of bias, and question two containing two options, with only option *a* scoring for low risk of bias. The answers selected in response to the seven questions were determined by two independent researchers (SCH, CM):

**Selection:**

1. Is the case definition adequate?
2. Yes, with independent validation (i.e., DSM/ICD Diagnosis) *
3. Yes, e.g., record linkage or based on self-reports (e.g., EDI-Q/MINI)
4. No description
5. Representativeness of the cases
6. Consecutive or obviously representative series of cases (female, BMI<18.5 or $\geq18.5- \leq25 for RecAN$)*
7. Potential for selection biases or not stated (e.g., sampling bias, high attrition/drop-out rate, underpowered sampling, partitioning of sample).
8. Selection of Controls
9. Community control*
10. Hospital controls
11. No description
12. Definition of Controls
13. No history of disease/current neurological, psychiatric or ED diagnosis.
14. No description of source

**Comparability:**

1. Comparability of cases and controls on the basis of the design or analysis
2. Study controls (female, healthy BMI>18.5 - <25, depression, and anxiety)*
3. Study controls for any additional factor (aged, education/IQ)*

**Exposure:**

1. Ascertainment of exposure
2. Standardised methodology (i.e., across subject counterbalancing of experimental conditions; repeated-measures design, pseudo-randomisation of experimental condition; between-subject design, appropriate control condition)*
3. Blinding of participants to condition(s), double-blinded for tasks that require experimenter scoring or tasks incorporating an intervention*
4. Experiment not blinded to case/control status
5. Self-report outcome responses/records only
6. No description
7. Same method of ascertainment for cases and controls
8. Yes*
9. No

**Social Attention:**

| Author  and Date |  | | | |  |  | |
| --- | --- | --- | --- | --- | --- | --- | --- |
|  | Selection | | | | Comparability | Exposure | |
|  | Adequacy of case  definition | Representativeness of cases | Selection of controls | Definition of controls | Comparability | Ascertainment of exposure | Same method of ascertainment for cases and controls |
| Bang et al., (2016) | a | a | a | a | a, b | a | a |
| Cardi et al., (2013) | a | b | a | a | a, b | a | a |
| Cserjesi et al., (2011) | a | a | a | a | a, b | a | a |
| Gilon Mann et al., (2018) | a | a | a | a | a | a | a |
| Goddard et al., (2014) | a | b | a | a | a, b | a | a |
| Harrison et al., (2010a) | a | a | a | a | a, b | a | a |
| Harrison et al., (2010b) | a | a | a | a | a, b | a | a |
| Kanakam et al., (2013) | a | b | a | a | a, b | a | a |
| Kim et al., (2014) | a | a | a | a | a, b | a, b | a |
| Manuel and Wade (2013) | b | b | a | b | *n/s* | a | a |
| Nuding et al., (2023) | a | a | a | a | *n/s* | a | a |
| Radix et al., (2023) | a | a | a | a | a | a | a |
| Schneier et al., (2016) | a | a | a | a | b | a | a |
| Schober et al., (2014) | a | a | a | a | a, b | a | a |
| Sfarlea et al., (2023) | a | a | a | a | a | a | a |

**Social Interpretation:**

| Author  and Date |  | | | |  |  | |
| --- | --- | --- | --- | --- | --- | --- | --- |
|  | Selection | | | | Comparability | Exposure | |
|  | Adequacy of case  definition | Representativeness of cases | Selection of controls | Definition of controls | Comparability | Ascertainment of exposure | Same method of ascertainment for cases and controls |
| An et al., (2023) | a | b | a | a | n/s | a | a |
| Cardi et al., (2017) | a | a | a | a | a | a | a |

**Social Memory Biases:**

| Author  and Date |  | | | |  |  | |
| --- | --- | --- | --- | --- | --- | --- | --- |
|  | Selection | | | | Comparability | Exposure | |
|  | Adequacy of case  definition | Representativeness of cases | Selection of controls | Definition of controls | Comparability | Ascertainment of exposure | Same method of ascertainment for cases and controls |
| Jänsch et al., (2009) | a | a | a | a | a, b | a | a |
| Manuel and Wade (2013) | b | b | a | b | *n/s* | a | a |
| Via et al., (2015) | a | a | a | a | a,b | a | a |

**Emotional Recognition and Emotional Regulation:**

| Author  and Date |  | | | |  |  | |
| --- | --- | --- | --- | --- | --- | --- | --- |
|  | Selection | | | | Comparability | Exposure | |
|  | Adequacy of case  definition | Representativeness of cases | Selection of controls | Definition of controls | Comparability | Ascertainment of exposure | Same method of ascertainment for cases and controls |
| Ambwani et al., (2016) | b | a, b | a | a | *n/s* | a | a |
| Dapelo et al., (2015) | a | a | a | a | b | a | a |
| Gramaglia et aal., (2016) | a | a | a | a | n/s | a | a |
| Jänsch et al., (2009) | a | a | a | a | a, b | a | a |
| Kessler et al., (2006) | a | a | a | a | b | a | a |
| Kucharska-Pietura et al., (2004) | a | a | a | a | b | a | a |
| Mendlewicz et al., (2005) | a | a | a | a | a, b | a | a |
| Pollatos et al., (2008) | a | a | a | a | a, b | a | a |
| Sfärlea et al., (2018) | a | a | a | a | a | a | a |
| Wyssen et al., (2019) | a | a | a | a | a | a | a |

**Affective, physiological, and behavioural response:**

| Author  and Date |  | | | |  |  | |
| --- | --- | --- | --- | --- | --- | --- | --- |
|  | Selection | | | | Comparability | Exposure | |
|  | Adequacy of case  definition | Representativeness of cases | Selection of controls | Definition of controls | Comparability | Ascertainment of exposure | Same method of ascertainment for cases and controls |
| Crucianelli et al., (2016) | a | a | a | a | a | a | a |
| Cartaud et al., (2024) | a | b | a | b | *n/s* | a | a |
| Het et al., (2014) | a | a | a | a | *n/s* | a | a |
| Meneguzzo et al., (2020) | a | b | a | a | *n/s* | a | a |
| Meneguzzo et al., 2023) | a | a | a | a | *n/s* | a | a |
| Miller et al., (2003) | a | a | a | a | *n/s* | a | a |
| Miller et al., (2009) | a | b | a | b | *n/s* | a | a |
| Monteleone et al., (2011) | a | a | a | a | a | a | a |
| Monteleone et al., (2018) | a | a | a | a | n/s | a | a |
| Monteleone et al., (2020) | a | a | a | a | n/s | a | a |
| Schmalbach et al., (2020) | a | b | a | a | n/s | a | a |
| Schmalbach et al., (2020a) | a | b | a | a | n/s | a | a |
| Schmalbach et al., (2020b) | a | b | a | a | n/s | a | a |
| Vaz-Leal et al., 2018) | a | a | a | a | a | a | a |
| Zonnevylle-Bender et al., (2005) | a | a | a | a | b | a | a |
